# Charge environments around phosphorylation sites in proteins

**DOI:** 10.1186/1472-6807-8-19

**Published:** 2008-03-25

**Authors:** James Kitchen, Rebecca E Saunders, Jim Warwicker

**Affiliations:** 1 Faculty of Life Sciences, University of Manchester, Michael Smith Building, Oxford Road, Manchester M13 9PT, UK; 2 High Throughput Screening, Cancer Research UK, 44 Lincoln's Inn Fields, London, WC2A 3PX, UK

## Abstract

**Background:**

Phosphorylation is a central feature in many biological processes. Structural analyses have identified the importance of charge-charge interactions, for example mediating phosphorylation-driven allosteric change and protein binding to phosphopeptides. Here, we examine computationally the prevalence of charge stabilisation around phosphorylated sites in the structural database, through comparison with locations that are not phosphorylated in the same structures.

**Results:**

A significant fraction of phosphorylated sites appear to be electrostatically stabilised, largely through interaction with sidechains. Some examples of stabilisation across a subunit interface are evident from calculations with biological units. When considering the immediately surrounding environment, in many cases favourable interactions are only apparent after conformational change that accompanies phosphorylation. A simple calculation of potential interactions at longer-range, applied to non-phosphorylated structures, recovers the separation exhibited by phosphorylated structures. In a study of sites in the Phospho.ELM dataset, for which structural annotation is provided by non-phosphorylated proteins, there is little separation of the known phospho-acceptor sites relative to background, even using the wider interaction radius. However, there are differences in the distributions of patch polarity for acceptor and background sites in the Phospho.ELM dataset.

**Conclusion:**

In this study, an easy to implement procedure is developed that could contribute to the identification of phospho-acceptor sites associated with charge-charge interactions and conformational change. Since the method gives information about potential anchoring interactions subsequent to phosphorylation, it could be combined with simulations that probe conformational change. Our analysis of the Phospho.ELM dataset also shows evidence for mediation of phosphorylation effects through (i) conformational change associated with making a solvent inaccessible phospho-acceptor site accessible, and (ii) modulation of protein-protein interactions.

## Background

The phosphoproteome is a window into key biological processes, such as gene expression and cell growth, that are mediated by kinase and phosphatase activities [1]. There is widespread interest in mediators of phosphorylation as therapeutic targets [2]. Studies of specific phosphorylation pathways are being supplemented with measurements from the developing field of phosphoproteomics [3,4], indicating that around one third of proteins may be phosphorylated in a given cellular snapshot [5]. A number of general databases are being developed to collate these data, such as Phospho.ELM (originally PhosphoBase) [6], the Phosphorylation Site Database [7], and PhosphoSite [8]. Some resources are linked to certain streams of data, such as plant phosphorylation (PlantsP [9]) and specific phosphoproteome experiments (Phosida [10]). Other databases, such as dbPTM [11], collect phosphorylations along with other post-translational modifications.

Phosphorylation site databases serve as a reference for construction of methods aimed at sequence-based prediction of phosphorylation sites. A range of machine-learning techniques have been used, including neural networks (NetPhos [12,13]), hidden Markov models (KinasePhos 1.0 [14,15]), support vector machines (PredPhospho and KinasePhos 2.0 [16-18]), Bayesian decision theory (PPSP [19]), and other forms of sequence profile analysis (Scansite 2.0, Phosite, GPS [20-22]). Such predictors perform particularly well when kinase specificities are taken into account [18], or when species-dependence is included [12]. Experimental studies of phosphorylation in yeast are consistent with strong preferences of kinases for specific substrates, but also indicate that predictions based on phosphorylation site patterns can lead to substantial over-prediction, and the conclusion that many sequences may not be accessible and that other regions may be involved in substrate recognition [23]. The current set of phosphorylation site databases and prediction servers has been recently reviewed [24].

Whilst it is clear that amino acid sequence and kinase-specific features play key roles in many phosphorylations, a number of studies have also looked at 3D structural encoding of features around phosphorylation sites. Local structure around phosphorylated and non-phosphorylated sites was used, in the form of contact maps, to train a neural network [13]. An algorithm to predict local structural segments from sequence has been used to enhance prediction accuracy for phosphorylation sites [25]. A database of 3D structures of protein phosphorylation sites has been developed, including structural annotation of the Phospho.ELM database (Phospho3D [26]). It has been noticed that the amino acid sequence properties around phosphorylation sites are similar to those of intrinsically disordered protein regions, and this information used to construct a prediction algorithm, DISPHOS [27]. Further work has demonstrated that intrinsic disorder is a characteristic of partners that bind 14-3-3 proteins [28], and that it may be a general feature of linear motifs that determine protein-protein interactions [29]. A survey of proteins for which structures are known in the phosphorylated and non-phosphorylated forms found that the effect of phosphorylation is mediated in many of these cases by conformational change, and by alteration of interfacial properties in some others [30].

Charge interactions have been investigated around subsets of phosphorylation sites, in phosphopeptide binding systems [31], at interfaces mediated by phosphorylated sites in general [32], and around activation loops containing phosphorylation sites [33,34]. In all of these cases, favourable charge-charge interactions were identified at the phosphorylated sites. For the phosphopeptide binding systems this is the major feature in a prediction tool [31], whilst complementary charge interactions drive the successful modelling of changes in activation loop conformation [34].

Model peptide studies have shown that phosphorylation stabilises α-helix formation when located at the amino terminus [35], consistent with calculations and the location of phosphate or sulphate ions in crystal structures [36]. Other work has highlighted the strength of non-covalent interactions that can be mediated by phosphorylated groups, salt-bridging in a model peptide [37], or mediating protein-protein interactions [38]. Calculations have been used to estimate the relative strengths of hydrogen bonds that involve phosphorylated amino acid sidechains [39]. We have previously used Finite Difference Poisson-Boltzmann (FDPB) calculations of charge interactions in studies of peptide phosphorylation [35]. The current study uses phosphorylated proteins in the structural database to address the question of whether phosphorylation sites can be distinguished in terms of charge interactions, relative to their non-phosphorylated counterparts within the same proteins. We find a subset of stabilised phosphorylated sites, many of which are within kinases, and for which the favourable interaction is derived largely from interactions with basic sidechains. When studied in the context protein structures that are not phosphorylated at these sites, an indication of favourable interactions (and conformational change) can be recovered by analysing the charge environment beyond the nearest neighbours. In an extension to phosphorylation sites from the Phospho.ELM database [6] that can be structurally annotated, we find that these do not have as large a signal indicative of conformational change as those from the structural database. On the other hand, the Phospho.ELM sites are more buried on average than the set derived from phosphorylated protein structures. There is also an interesting difference in the distributions of patch polarity around Phospho.ELM sites and background sites.

## Results and discussion

### Phosphorylated proteins in the structural database

**Figure 1:**
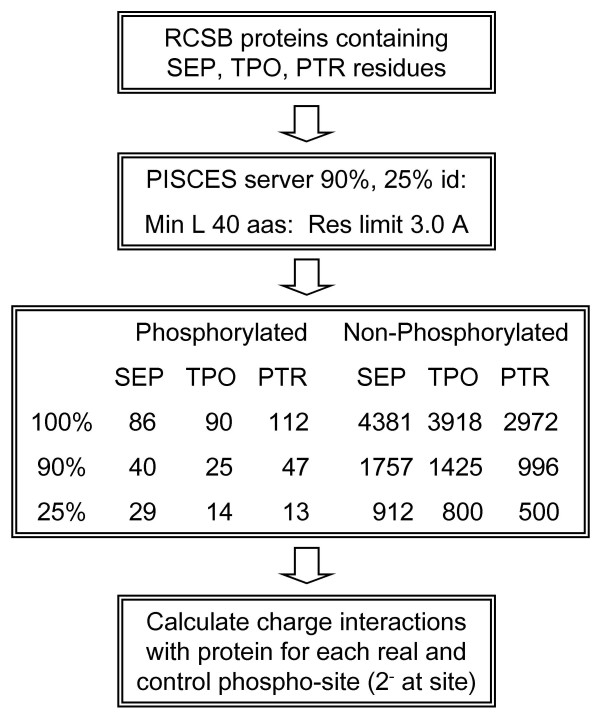
Scheme for collecting proteins from the PDB, that are phosphorylated on serine, threonine or tyrosine sidechains, using phosphorylated residue identifiers, Sep, Tpo or Ptr, respectively.

Figure 1 shows the scheme for extracting proteins phosphorylated on Serine (Sep), Threonine (Tpo) or Tyrosine (Ptr) sidechains, from the Protein Data Bank (PDB [40]). Phosphorylated sets were made by reduction according to sequence identity at 100%, 90% and 25% levels, using the PISCES server [41]. In order to compare phosphorylated and non-phosphorylated sites, without model-building torsional angles for phosphate groups on the non-phosphorylated sites, phosphate groups were removed from the phosphorylated sites. Calculations of charge interactions were then made between a notional -2e charge on the OG, OG1 and OH atoms (Ser, Thr and Tyr), and the surrounding environment. Interactions were therefore equivalent in terms of acceptor site geometry for phosphorylated and non-phosphorylated sidechains.

**Figure 2:**
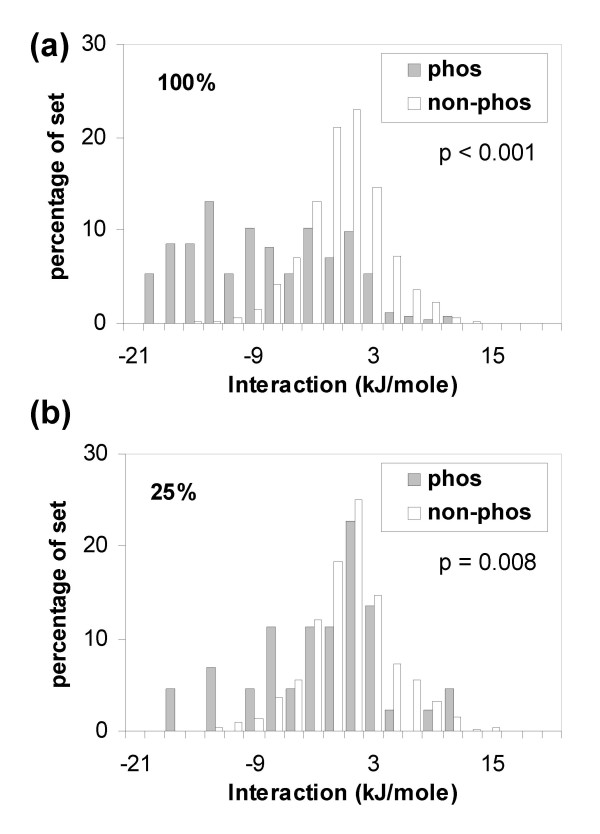
**Charge interactions of (Ser, Thr, Tyr) phospho-acceptor sites.** Distributions are compared for sites that are phosphorylated in the PDB (phos) and those that are not phosphorylated (non-phos) in the same coordinate files. In these, and succeeding phos/non-phos plots, probability (p) values of the phos and non-phos distributions arising from the same underlying population were estimated with the Mann-Whitney U test. (a) 100%, no removal of sequences. (b) Sequences culled at 25% sequence identity.

**Figure 3:**
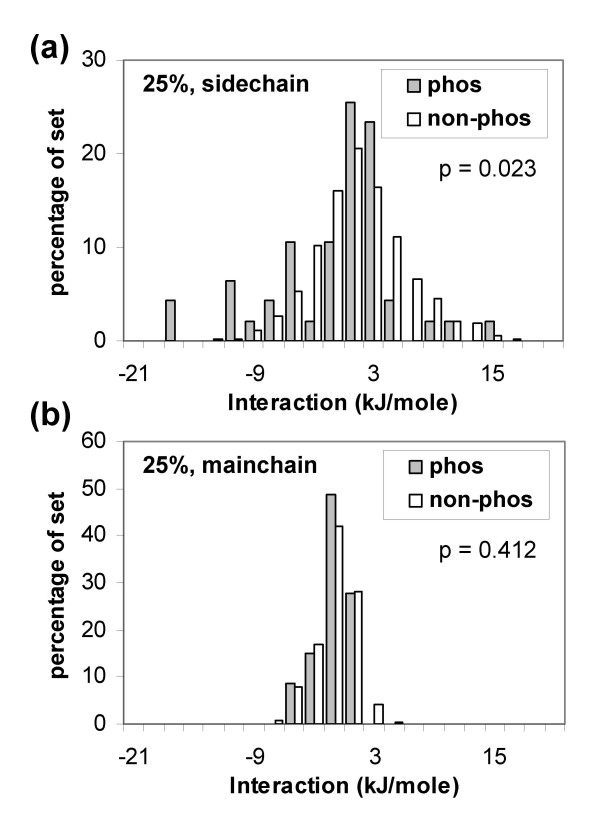
Charge interactions for (Ser, Thr, Tyr) acceptor sides divided according to interactions with surrounding sidechain (a) or mainchain (b) groups.

Figure 2 shows results for the 100% and 25% sequence identity-culled sets. Separation between the distributions is evident for both sets. Figure 3 shows, for the non-redundant set at 25% sequence identity, the relative importance of interactions from surrounding mainchain and sidechain components. The major determinant of stabilisation is (basic) sidechain interaction with phosphorylated sites. These basic sidechains often, but not exclusively, occur within the local sequence neighbourhood of the phosphorylated site, as is the case for substrates of basophilic protein kinases. Favourable interaction with sidechains contrasts to the relatively well-known occurrences of sulphate ion stabilisation at an α-helix terminus in protein crystal structures [42], and with the role of P-loop mainchain interaction with the phosphates of ATP and GTP [43]. Mainchain interactions may be underestimated relative to sidechain interactions, since our model omits specific hydrogen-bonds associated with phosphate oxygens, concentrating on net charge.

**Figure 4:**
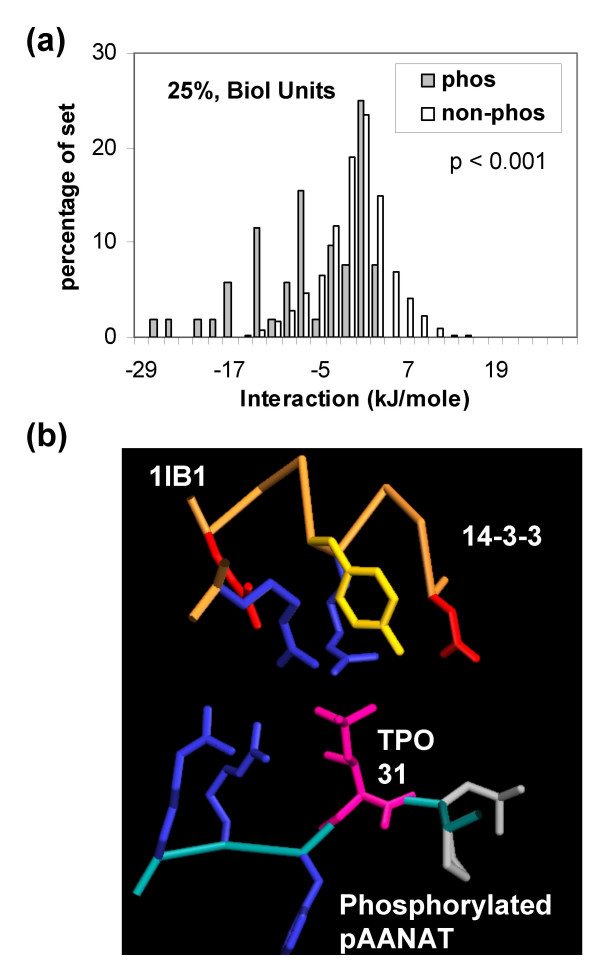
**Charge stabilisation of phosphorylated sites in biological units.** (a) Phosphorylated and background sites in the dataset culled at 25% sequence identity. (b) Phosphorylation site stabilisation at a protein-protein interface. An extended segment of phosphorylated serotonin N-acetyltransferase (AANAT) (mainchain, cyan and TPO 31, pink) binds to 14-3-3 (orange mainchain), with surrounding residues colour-coded by type (blue basic, red acidic, yellow polar uncharged, grey non-polar).

We examined the effect of replacing single protein chains with biological units (i.e. physiologically relevant oligomers, Figure 4a). The results are similar to those for the single chains (Figure 3), but with greater separation of the phosphorylated and non-phosphorylated sets, indicative of some phosphate-mediated stabilisation between protein chains. Figure 4b shows an example, where the binding of phosphorylated serotonin N-acetyltransferase and isoform ζ of 14-3-3, 1ib1 [44], is analagous to the phosphopeptide binding complexes, in which charge interactions were observed to be a key feature of binding [31]. The majority of these systems were excluded through the use of a minimum polypeptide length of 40 amino acids. Other examples of stabilisation between protein chains also relate to segments that would be unstructured in the absence of phosphorylation: β-catenin repeat interactions with both E-cadherin, 1i7w [45], and phosphorylated APC, 1v18 [46], and the forkhead-associated domain of Ki67 with a phosphorylated fragment of human nucleolar protein, 2aff [47].

Many of the structures studied show little stabilisation, or show destabilisation, in our estimate of charge-charge interactions. In a few instances, small overall interaction results from near cancelling of stabilising and destabilising contributions, as at the phosphorylated active site of phosphoglucomutase, 1vkl [48], with opposing contributions from a metal ion and its ligands. In a larger number of cases, the surrounding charge interactions are generally small. Some of these at least may be indicative of phosphorylation mediating differences in the formation of higher order structures or complexes that are not included in the current analysis. Examples are the surface properties and fibre formation in pilin, 2pil [49], and the phosphorylation of nucleotide-binding domain 1 and potential interactions with other components of the cystic fibrosis transmembrane conductance regulator, 1r0z [50]. As more structures become available for complexes, and using comparative models built on protein-protein interaction templates [51], a more thorough study of the extent to which phosphorylation mediates interfacial properties will be possible.

For the PDB set, non-redundant at 25% sequence identity, and using a threshold interaction of -6 kJ/mole, the current method gives (sensitivity, specificity) of (0.48, 0.90), compared with (0.52, 0.67) for NetPhos [13] and (0.65, 0.79) for DISPHOS [27], using the default thresholds.

### Phosphorylation sites in Phospho.ELM

**Figure 5:**
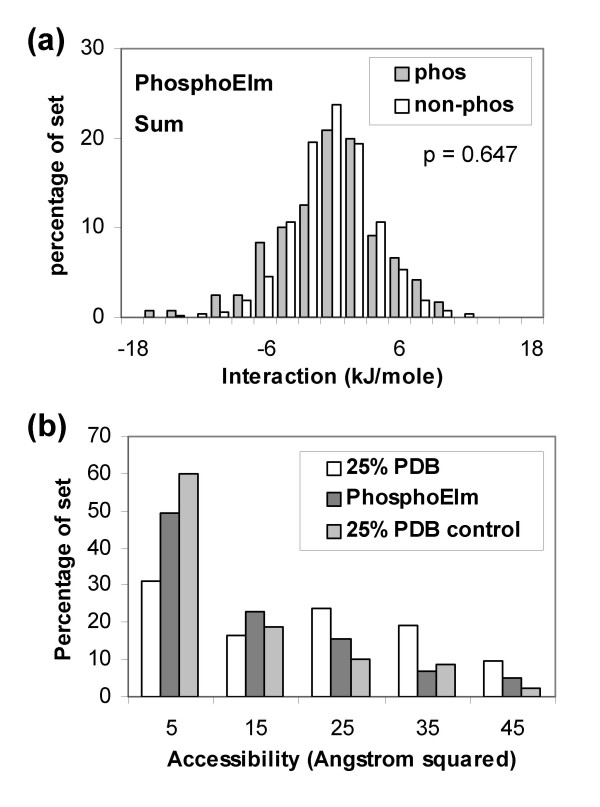
**Calculations with structurally annotated phosphorylation sites from the Phospho.**ELM dataset. (a) Summed charge interactions. (b) Solvent accessibility for phospho-acceptor atoms in the 25% sequence identity culled PDB set, in the Phospho.ELM set, and in a control set (other Ser, Thr, Tyr in the 25% PDB set).

A scheme was employed to structurally annotate phosphorylated sequences contained in the Phospho.ELM dataset (see Methods). Figure 5a shows that little separation is apparent between the phosphorylated sites and other Ser, Thr, Tyr locations in the same set. Proteins identified in this structural annotation are generally non-phosphorylated. Considering possible structural adaptation to phosphorylation, the most simple to model would be movement of sidechains on a fixed backbone framework. Thus, in the absence of the negative charge of a phosphorylated group binding to a basic patch, an alternative acidic sidechain could occupy a similar location. In order to test whether this simple picture would aid distinction of phospho-acceptor sites, we calculated interactions with positively-charged residues only. Again no separation is apparent (not shown). In terms of (sensitivity, specificity), the current method, using the -6 kJ/mole threshold, gives (0.15, 0.92), compared with (0.66, 0.68) for NetPhos [13] and (0.64, 0.77) for DISPHOS [27]. These results emphasise how poorly the present charge-based algorithm performs for the Phospho.ELM set, in contrast to the PDB set, leading us to consider other features that could mediate the effects of phosphorylation.

Solvent accessibility of phosphorylation sites could be an important factor, although conformational change will again play a role. Figure 5b shows that phosphorylated sites in the PDB set that is non-redundant at 25% sequence identity, are on average more accessible than the control set. Interestingly, phosphorylated sites from the structurally annotated Phospho.ELM set appear to be more buried, on average, than do those from the 25% PDB set. Both sets of calculations displayed in Figure 5b were made with single protein chains, rather than biological units, and should therefore be comparable.

**Figure 6:**
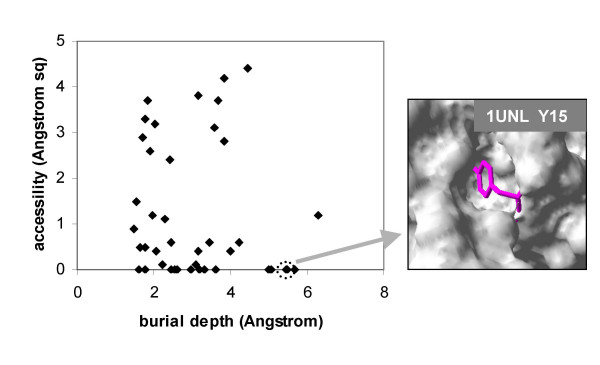
**Burial depth (from exterior solvent surface) of Phospho.ELM sites that have low solvent accessibility.** Scatter plot of burial depth (see text) against accessibility for all sites with ASA < 5 Å^2^, with a surface depiction of the environment around a buried tyrosine phospho-acceptor site in PDB file 1unl.

We examined some of the most buried sites in the structurally annotated Phospho.ELM dataset. In order to probe the burial in more detail, a burial depth was calculated from exterior solvent (see the Methods section), for the least solvent accessible sites (0 – 5 Å^2 ^ASA, Figure 6). This is a grid-based method, with a 2 Å burial simply due to van der Waals radius, and about +/- 0.5 Å due to the grid spacing. We find that many sites lie at a burial depth of around 2 Å, effectively at the exterior surface. Even at larger burial depth, we find sites with some solvent accessibility, i.e. solvent pockets within a protein. Of particular interest are the sites that are most buried, according to our algorithm, with least accessibility. Amongst such sites, at zero calculated solvent accessibility, we see known examples of phosphorylation control of function, e.g. Y15 in cyclin-dependent kinase, 1unl, shown in Figure 6, [52]; Y19 in the adipocyte lipid-binding protein, 1a18, [53]; and S579 in human plasmin, 1bml, [54]. In each of these cases the structure from our annotation procedure is inconsistent with phosphorylation (zero solvent accessibility and substantial burial), indicative of conformational change. Although the effects of phosphorylation in these Phospho.ELM entries are generally well-characterised, this work demonstrates the possibility for prediction of conformational change in less well-studied examples, such as those emerging from mass spectrometry data.

### Phosphorylated and non-phosphorylated structures

**Figure 7:**
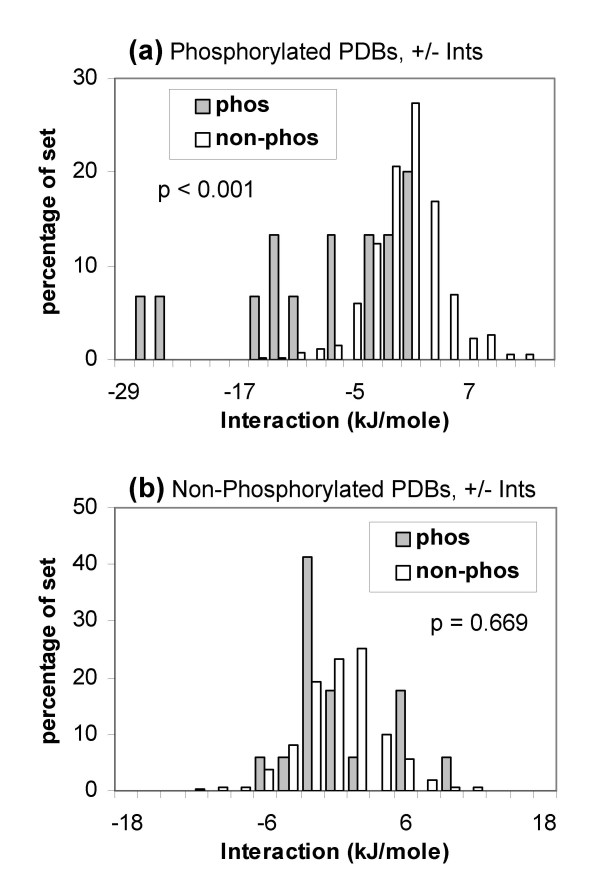
**Comparison of structure-based calculations for proteins with phosphorylated and non-phosphorylated structures.** (a) Charge interactions of the phos and non-phos sites in coordinate files that are phosphorylated. (b) The equivalent sites to panel (a), in coordinate files that are not phosphorylated.

Figure 7 presents a comparison of sites from the PDB set, for which resolved structures exist in both phosphorylated and non-phosphorylated states. It is evident that whilst there is separation of the phosphorylated sites (from other Ser, Thr, Tyr), this is not the case for the equivalent non-phosphorylated sites. Thus conformational change between ordered segments leads to charge stabilisation in these systems. This result is consistent with our study of the Phospho.ELM dataset, where known phospho-acceptor sites do not exhibit charge stabilisation when studied in the context of non-phosphorylated structures.

### Potential peaks around phospho-acceptor sites

**Figure 8:**
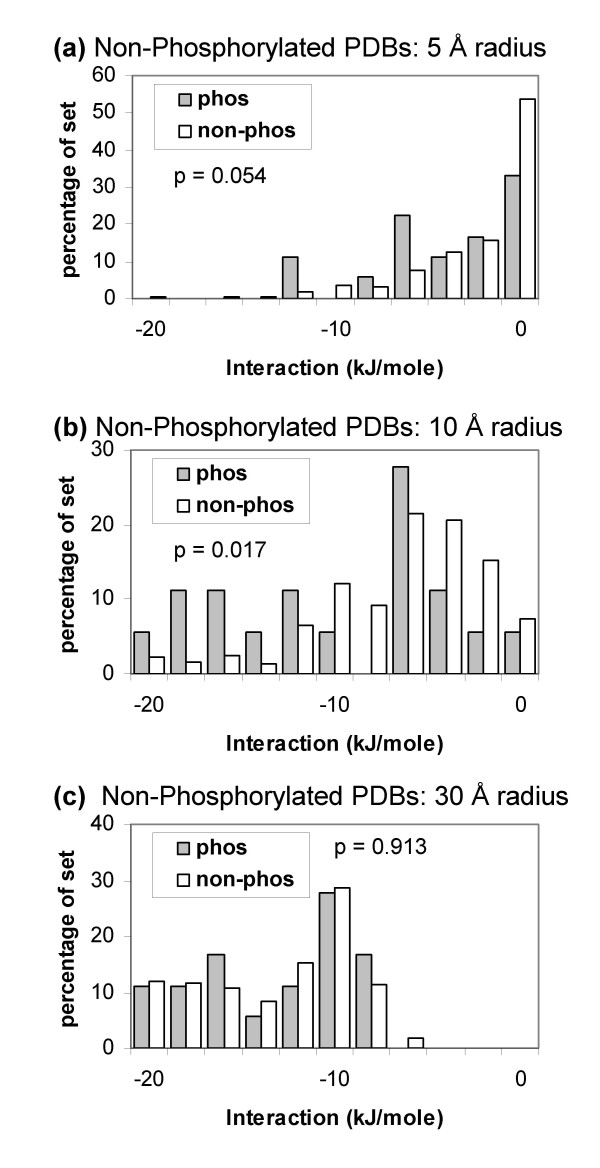
**Searching for favourable interactions around phospho-acceptor sites in proteins that are not phosphorylated.** Calculations were made with the non-phosphorylated set of proteins, from the phosphorylated/non-phosphorylated structure pairs, many of which undergo conformational change upon phosphorylation. A search was made, within a given radius, for the most positive potential peak around each site (real/phos or background/non-phos). Interaction energy is given for a single unit negative charge in the positive potential field. (a) Radius = 5 Å. (b) Radius = 10 Å. (c) Radius = 30 Å.

Since mainchain conformational change plays a role in some phosphorylations, we adapted previous work that studied electrostatic potential values on a solvent accessible shell [55]. In this case we are looking for the most favourable interacting site (highest positive potential) within a given radius of each site (centred on Ser OG, Thr OG1 or Tyr OH). Figure 8 shows this analysis for the non-phosphorylated coordinate sets of the previous section, at three radii. At 30 Å radius, the interaction values are generally favourable and there is little separation between the real and background sets, since the large search radius links all Ser, Thr, Tyr sites to favourable patches for charge interaction. Of more interest are the smaller radii. Some separation is apparent at 5 Å radius, which increases at 10 Å radius, and is largely associated with the proteins (including kinases) that underpin separation in earlier Figures (e.g. Figure 7a). The procedure of electrostatic peak finding within a sphere around a phospho-acceptor site, but for non-phosphorylated molecules, largely recovers the result of calculations with phosphorylated coordinate sets. This occurs without knowledge of the loops that undergo conformational change. The method could therefore aid identification of such regions, and allied with simulation methods [34], provide structural models for conformational change that is coupled to phosphorylation.

**Figure 9:**
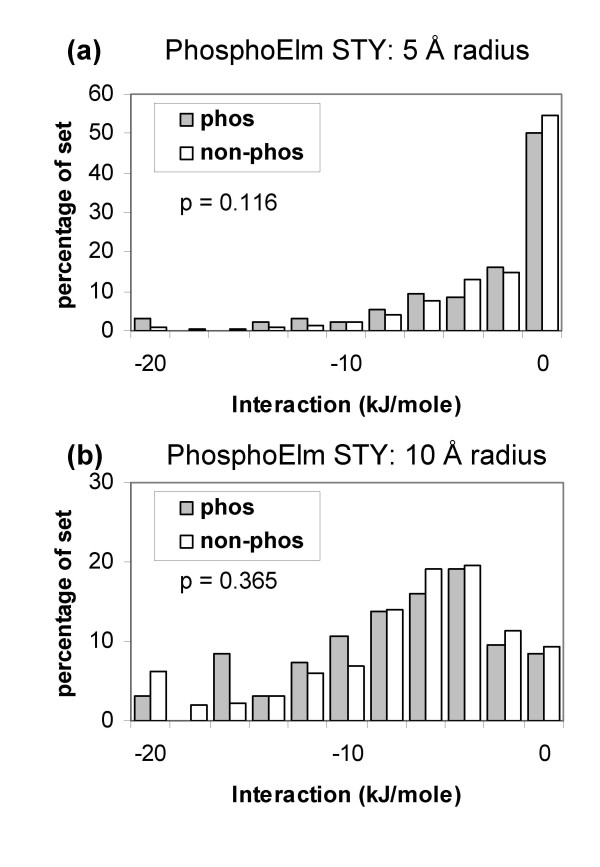
**Favourable interactions around Phospho.ELM sites compared with background sites.** Structural annotation was made with non-phosphorylated coordinate files. (a) Maximal charge interactions around structurally annotated Ser, Thr and Tyr sites at 5 Å radius. (b) Maximal interactions around Ser, Thr, Tyr sites at 10 Å radius.

The same, patch-based, calculation was made for the 101 members of the Phospho.ELM set that could be structurally annotated (Figure 9). Separation of the Ser, Thr, Tyr phos from non-phos sets is small at both the radii shown.

**Figure 10:**
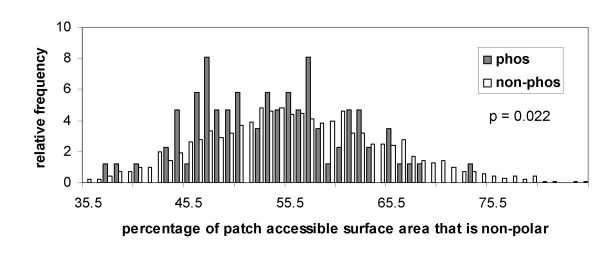
**Polarity of patches around phos and non-phos sites in the structurally annotated Phospho.**ELM dataset. Distributions are plotted for surface patches consisting of residues of ASA > 5 Å^2^, within 10 Å of a central Ser, Thr, Tyr.

The patch search method appears able to identify some regions that are susceptible to phosphorylation/charge-driven conformational change, and will be studied in more detail in future work, both with conformationally characterised systems and with high-throughput proteomics data. However, there also many proteins for which charge-charge effects are not clearly apparent in transducing phosphorylation activity, particularly in the Phospho.ELM set. In some cases, it is possible that low solvent accessibility is important (Figure 5b), and in others complexation may play a role. In a preliminary analysis of this last factor, we calculated the non-polar percentage of ASA in patches around phos and non-phos sites for the structurally annotated Phospho.ELM dataset (Figure 10). The distributions are different, with a bimodal shape for the phos sites and more unimodal for the non-phos subset. It is not clear at this stage what underpins this difference, but one possibility is that the effects of phosphorylation for many of these proteins could be mediated via interfacial regions in protein-protein interactions.

## Conclusion

We have studied the charge environments around phospho-acceptor sites in phosphorylated and non-phosphorylated protein structures, and extended the study to structurally annotated sites in Phospho.ELM. Our results show that only a subset of phosphorylated structures (around a third in our analysis of the PDB) appear to be making use of particularly favourable charge interactions at the site of phosphorylation, when considered in the context of biological unit. Several of these examples are protein kinases with phosphorylated activation loops. When non-phosphorylated structures are studied, much of the signal of excess favourable charge interactions relative to a control set is lost, consistent with a degree of conformational change (e.g. the activation loop of protein kinases). We introduce a simple algorithm, using the charge/electrostatic potential neighbourhood, to recover the signal for non-phosphorylated structures. This method has the potential to aid studies of conformational change associated with phosphorylation, since it predicts the target area for phosphate binding. The result is consistent with molecular simulations in which charge interaction was seen to be the key factor in establishing an anchor point for phosphorylated residues [34].

For a set of phosphorylated sites structurally annotated from Phospho.ELM, we find little signal for charge stabilisation around phospho-acceptor sites, even when the neighbourhood algorithm is applied. This result suggests that any phosphorylation-induced conformational change is relatively complex or that the effects of phosphorylation are mediated in other ways, for many of the Phospho.ELM proteins. We find indirect evidence in both of these directions. The Phospho.ELM set has a tendency towards buried sites (in non-phosphorylated forms), possibly indicative of substantial conformational change. A preliminary analysis of patch surface polarity in the Phospho.ELM set shows a different distribution for phos sites relative to non-phos sites, suggesting that complexation could play a role i.e. where phosphorylation alters the propensity for forming an interface.

Further work will study phosphorylation sites in the context of protein-protein interaction databases (e.g. PIBASE, [56]). There are also issues of detailed modelling, such as inclusion of phosphate group torsion angles, emphasising phosphate interactions with mainchain groups, and adding Asp and His sidechains as acceptors. Our longer term aims are to probe molecular mechanisms by which phosphorylation mediates biological effects, and improve predictive algorithms that are based on sequence properties, using for example kinase specificity or disorder [12,27]. The issue of disorder is of particular interest since our structural annotation of Phospho.ELM loses many phosphorylated sites as unstructured regions within known folds. It will be of interest to extend our patch algorithm to such examples, given that we also find examples in the structural database where sites that are ordered when phosphorylated, are unstructured when non-phosphorylated.

## Methods

### PDB datasets

Structures containing as ligand type Sep, Tpo or Ptr representing phosphoserine, phosphothreonine and phosphotyrosine respectively, were identified in the PDB [40]. Single chains were selected to remove redundant copies of phosphorylated sites within a coordinate file. Redundancy between PDB sets was investigated using the PISCES server [41], selecting crystal structures with a minimum polypeptide length of 40 amino acids, and resolution better than 3.0 Å. We carried through sets of phosphorylation sites at 100%, 90% and 25% non-redundancy, based on sequence identity. These sets represent the complete phosphorylation data (100%), removal of near-identical copies (90%), and removal of copies that are clearly homologous (25%). This scheme is shown in Figure 1, with the numbers of phosphorylated sites retrieved. In addition to the phosphorylated sites, other Ser, Thr and Tyr residues in the same proteins constituted a non-phosphorylated control set, which was submitted to the same calculations. Generally for any set of PDB files containing phosphorylated sites, the control set was derived from that set of proteins, although it is possible that some of these other sites could be phosphorylated under different conditions. For calculations with biological units, the relevant coordinate files were obtained from the PDB.

An additional dataset was formed by searching for non-phosphorylated structures that are related PDB entries to the phosphorylated structures, with the caveat that the site of phosphorylation should be ordered in both cases. This dataset was derived from the 90% sequence identity culled set of phosphorylated coordinate files.

### Phospho.ELM dataset

Phosphorylation sites from the Phospho.ELM database were also studied, using version 5.0, released May 2006 [6]. The approximately 7000 instances of phosphorylated serine, threonine and tyrosine were reduced to just 118 PDB files, with our structural annotation procedure.

### Structural annotation of sequences

The UNIPROT protein sequence database was used to underpin the structural annotation process [57]. Phosphoprotein accession numbers from Phospho.ELM were matched to UNIPROT, and then to 3D structures where available. Phosphopeptide sequences extracted from Phospho.ELM were matched to the amino acid sequence present in the coordinate section of the PDB file for verification. Where a phosphopeptide sequence matches to more than one PDB file, the first match was used.

### Charge calculations

Charge interactions are compared at phosphorylated and non-phosphorylated Ser, Thr, Tyr sites. This is achieved in a model where a notional -2e charge is placed on the OG atom of Ser, OG1 of Thr and OH of Tyr. For this purpose, the phosphate group is removed from any phosphorylated residue, allowing direct comparison with non-phosphorylated residues. There are number of approximations in this process, notably the lack of phosphate group modelling and the -2e charge. The pKas of phosphorylated sidechains for the -1e to -2e transition suggest that the -2e state will predominate at neutral pH [35]. Nevertheless, our focus is on comparison of phosphorylated and non-phosphorylated residues, rather than absolute values. The same rationale underlies the omission of a phosphate group model. To maintain direct comparisons, without removing phosphates, these groups would need to be modelled on non-phosphorylated residues, and results averaged over (or selected from) torsional variation.

The Debye-Hückel (DH) model for charge-charge interactions is used, with a relative dielectric (78.4) corresponding to water, and an ionic strength of 0.15 Molar. This model does not account for charge interactions with the less polarisable protein, but it generally works well for charge-charge interactions at a protein surface [58], which are the current target. We compared the DH model with Finite Difference Poisson-Boltzmann calculations of charge-charge interactions. The relative distributions of charge interaction energies for phosphorylated and non-phosporylated sites were similar to those for the DH model (not shown).

Distributions are displayed as histograms of interaction energy, summed over all phosphorylated (phos) or non-phosphorylated (non-phos) residues in the coordinate files of a dataset. Percentages are plotted, facilitating comparison of the unequal-sized datasets. The Mann-Whitney U test is applied to the series of interaction values for each phos and non-phos set. The probability of occurrence of these series, if there is no difference in the underlying distributions, is given for each phos/non-phos plot. A significant difference is generally inferred at the 5% (p < 0.05) level.

### Solvent accessibility and burial depth

Solvent accessibility was calculated for phospho-acceptor atoms, OG (Ser), OG1 (Thr), OH (Tyr), using an in-house program, and the data were binned for histogram display. Burial depth from the exterior solvent surface was calculated with a grid-based algorithm. Molecule points are assigned on the grid for regions that are not solvent accessible. Then starting from outside of the molecule, a grid of accessibility to the exterior solvent is constructed. The burial distance (to the exterior surface) is calculated between each atom and the nearest exterior accessible grid point. A value of around 2 Å represents exterior surface located, taking into account the united atom van der Waals radius set.

### Polarity of surface patches in the Phospho.ELM dataset

The surface polarity around Ser, Thr and Tyr residues was calculated for amino acids within a 10 Å radius (based on C_α _atoms), and with ASA > 5 Å^2^. Distributions of non-polar percentage (i.e. 100 × non-polar area/total area) were compared between phos and non-phos sites.

## Availability and requirements

Project name: Phospatch

Project home page: 

The software used in this study is also available for download from: 

Operating system(s): Linux

Programming language: Perl, Fortran

License: GNU GPL

No additional restrictions for non-academic users.

## Abbreviations

DH, Debye-Hűckel; FDPB, Finite Difference Poisson-Boltzmann; PDB, Protein Data Bank; ASA, Accessible Surface Area; ATP, Adenosine 5'-triphosphate; GTP, Guanosine 5'-triphosphate; 3D, 3-Dimensional.

## Authors' contributions

RES and JW carried out an initial study. JK and JW planned and performed subsequent work, and wrote the manuscript. All authors read and approved the final manuscript.
